# Multiscale modeling of HBV infection integrating intra- and intercellular viral propagation to analyze extracellular viral markers

**DOI:** 10.1371/journal.pcbi.1011238

**Published:** 2024-03-11

**Authors:** Kosaku Kitagawa, Kwang Su Kim, Masashi Iwamoto, Sanae Hayashi, Hyeongki Park, Takara Nishiyama, Naotoshi Nakamura, Yasuhisa Fujita, Shinji Nakaoka, Kazuyuki Aihara, Alan S. Perelson, Lena Allweiss, Maura Dandri, Koichi Watashi, Yasuhito Tanaka, Shingo Iwami

**Affiliations:** 1 interdisciplinary Biology Laboratory (iBLab), Division of Natural Science, Graduate School of Science, Nagoya University, Nagoya, Japan; 2 Department of Scientific Computing, Pukyong National University, Busan, South Korea; 3 Department of Virology II, National Institute of Infectious Diseases, Tokyo, Japan; 4 Department of Gastroenterology and Hepatology, Faculty of Life Sciences, Kumamoto University, Kumamoto, Japan; 5 Faculty of Advanced Life Science, Hokkaido University, Sapporo, Japan; 6 International Research Center for Neurointelligence, The University of Tokyo Institutes for Advanced Study, The University of Tokyo, Tokyo, Japan; 7 Theoretical Biology and Biophysics Group, Los Alamos National Laboratory, Los Alamos, United States of America; 8 Department of Internal Medicine, University Medical Center Hamburg-Eppendorf, Hamburg, Germany; 9 German Center for Infection Research (DZIF), Hamburg-Lübeck-Borstel-Riems partner sites, Germany; 10 Research Center for Drug and Vaccine Development, National Institute of Infectious Diseases, Tokyo, Japan; 11 Department of Applied Biological Sciences, Faculty of Science and Technology, Tokyo University of Sciences, Chiba, Japan; 12 Institute of Mathematics for Industry, Kyushu University; Fukuoka, Japan; 13 Institute for the Advanced Study of Human Biology (ASHBi), Kyoto University; Kyoto, Japan; 14 NEXT-Ganken Program, Japanese Foundation for Cancer Research (JFCR), Tokyo, Japan; 15 Interdisciplinary Theoretical and Mathematical Sciences (iTHEMS), RIKEN, Wako, Japan; 16 Science Groove Inc., Fukuoka, Japan; University of Illinois at Chicago, UNITED STATES

## Abstract

Chronic infection with hepatitis B virus (HBV) is caused by the persistence of closed circular DNA (cccDNA) in the nucleus of infected hepatocytes. Despite available therapeutic anti-HBV agents, eliminating the cccDNA remains challenging. Thus, quantifying and understanding the dynamics of cccDNA are essential for developing effective treatment strategies and new drugs. However, such study requires repeated liver biopsy to measure the intrahepatic cccDNA, which is basically not accepted because liver biopsy is potentially morbid and not common during hepatitis B treatment. We here aimed to develop a noninvasive method for quantifying cccDNA in the liver using surrogate markers in peripheral blood. We constructed a multiscale mathematical model that explicitly incorporates both intracellular and intercellular HBV infection processes. The model, based on age-structured partial differential equations, integrates experimental data from in vitro and in vivo investigations. By applying this model, we roughly predicted the amount and dynamics of intrahepatic cccDNA within a certain range using specific viral markers in serum samples, including HBV DNA, HBsAg, HBeAg, and HBcrAg. Our study represents a significant step towards advancing the understanding of chronic HBV infection. The noninvasive quantification of cccDNA using our proposed method holds promise for improving clinical analyses and treatment strategies. By comprehensively describing the interactions of all components involved in HBV infection, our multiscale mathematical model provides a valuable framework for further research and the development of targeted interventions.

## Author summary

In viral infectious diseases, evaluating the quantity and dynamics of the virus within target cells is crucial for understanding disease progression caused by the virus and predicting the efficacy of anti-viral drugs. HBV, which infects the human liver, increases the risk of development of hepatocellular carcinoma with chronic infection. To avoid the risk, viral elimination by anti-viral treatment is needed. The final goal of HBV treatment is the elimination of cccDNA known as a viral reservoir, although it is challenging with current treatment. One of the barriers to this lies in the fact that cccDNA remains within the liver without entering the peripheral blood, making it difficult to assess the quantity and dynamics of cccDNA within the patient. To quantitate cccDNA, an invasive method called liver biopsy is necessary. This method involves inserting a large needle through the abdomen into the liver, which is basically not accepted. In this study, we constructed a multiscale mathematical model based on the experimental data, which allowed us to evaluate the quantity and dynamics of cccDNA using viral markers in the peripheral blood, if the baseline cccDNA amount prior to treatment has been quantified. The method we have developed is expected to significantly contribute to the improvement of future HBV treatment strategies.

## Introduction

Chronic hepatitis B virus (HBV) infection is a major public health problem, affecting approximately 297 million people worldwide (https://www.who.int/en/news-room/fact-sheets/detail/hepatitis-b), and increasing the likelihood of developing hepatocellular carcinoma. According to a World Health Organization (WHO) report, HBV infection caused the death of 820,000 people in 2019. Currently, pegylated interferon alpha (PEG IFN-α) and nucleos(t)ide analogues (NAs) are used as therapeutic agents for chronic hepatitis B [1]. PEG IFN-α suppresses viral replication by activating the host’s immune response, while NAs strongly reduce the amount of HBV DNA by inhibiting reverse transcription [2]. These treatments are effective in reducing viral load and thereby in improving hepatitis, but they are not curative, largely because of the persistence of covalently closed circular DNA (cccDNA), which is responsible for chronic hepatitis B, in many patients [3]. Therefore, to develop effective therapeutic strategies, we must first be able to quantify the amount of cccDNA and understand its dynamics. Liver biopsy is typically required to quantify cccDNA to assess its eradication, but this procedure is not commonly performed in clinical practice.

HBV infects host hepatocytes via binding to the viral receptor, sodium-taurocholate co-transporting polypeptide (NTCP), and is then transported to the nucleus to form cccDNA [4]. The cccDNA is a template for viral replication, which produces viral mRNAs with different lengths. One of the transcripts of approximately 3.5 kb in length, called pre-genomic (pg) RNA is reverse transcribed into HBV DNA. Additionally, the cccDNA stimulates the production of viral proteins such as HBV surface antigen (HBsAg) and HBV core-related antigen (HBcrAg). Integrated HBV DNA (iDNA) of host chromosomes also contributes to the presence of HBV antigens, particularly HBsAg [5,6]. HBsAg forms the viral envelope and is released to the serum as either an infectious particle with HBV DNA or a subviral particle. HBcrAg includes HBV core antigen (HBcAg), which forms a viral capsid, in addition to HBV e antigen (HBeAg) and a truncated core-related protein called p22cr as nonstructural viral proteins [7,8]. Thus, cccDNA in hepatocytes plays a crucial role in the persistence of HBV infection.

Mathematical modeling plays a crucial role in understanding the complex dynamics of viral infections [9–11]. In the case of HBV infection, mathematical models described by ordinary differential equations (ODEs) have been extensively used to investigate various aspects of the infection process [12,13]. By capturing the dynamics of intercellular HBV infection, these mathematical models have enabled us to quantify the reduction of HBV DNA during therapy [14–16]. The decline of HBV DNA can be accurately estimated by using ODEs to mimic the dynamics of intracellular HBV replication [12,13,17]. Moreover, a mathematical model has been employed to explore the role of HBsAg production from integrated DNA during HBV infection [13]. Through the use of clinical data, intercellular infection models can distinguish between the kinetics of the noncytolytic and cytolytic immune responses during acute HBV infection [18]. While mathematical models have been proposed that investigate the impact of HBeAg on immunological tolerance during HBV infection [19] and that include antibody response [20,21], further mathematical models are needed to elucidate the immune response [22]. In contrast to the multiscale models described by partial differential equations (PDEs) developed to study the effects of drugs on HCV infection, there is a notable absence of multiscale models for HBV infection that integrate intracellular and intercellular dynamics [23–25]. Furthermore, although the primary goal of therapy currently is to achieve functional cure with cccDNA inactivation, few studies have incorporated cccDNA, the molecular reservoir of HBV, into their mathematical models or linked the models to experimental data [17,22].

In this study, our aim was to devise a noninvasive method for quantifying intrahepatic cccDNA *in vivo*, employing surrogate markers present in peripheral blood. To achieve this goal, and to take advantage of describing the interactions of all components, we developed a multiscale mathematical model that explicitly incorporates both intracellular and intercellular HBV infection processes (e.g., [26]). Specifically, the in vitro experiments were conducted using primary human hepatocytes (PHH) for HBV infection. Cells treated with or without entecavir (ETV) were observed for up to 31 days post-infection. HBV infection was evaluated by detecting viral markers including intracellular and extracellular HBV DNA, as well as cccDNA, which were quantified by real-time PCR. These experiments were designed to make a simple intracellular HBV model, and we developed the multiscale model (i.e., age-structured PDEs) by integrating the intracellular model with a standard intercellular virus infection model. In the *in vivo* experiments using humanized mice, to track viral infection, we assessed viral markers such as HBsAg, HBeAg, HBcrAg, and HBV DNA in serum, and cccDNA in the liver, by ELISA, real-time PCR, and droplet digital PCR, respectively. By applying our multiscale mathematical model to the *in vivo* data sets, we roughly predicted the amount and dynamics of intrahepatic cccDNA within a certain range. This methodology, which quantifies serum viral markers, holds promise for advancing our understanding of chronic HBV infection and may pave the way for improved clinical analyses and treatment strategies.

## Results

**Fig 1:**
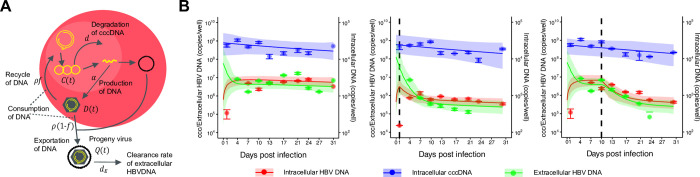
Dynamics of HBV infection in PHH cells. **(A)** Modeling of the intracellular viral life cycle in HBV-infected primary human hepatocytes is shown. Intracellular HBV DNA is produced from cccDNA at rate *α* and is consumed at rate *ρ*. That is, a fraction 1−*f* of HBV DNA assembled with viral proteins as virus particles is exported from infected cells, and the other fraction *f* is reused for further cccDNA formation having a degradation rate of *d*. **(B)** Fits of the mathematical model (solid lines) to the experimental data (filled circles) on intracellular HBV DNA and cccDNA and extracellular HBV DNA in PHH without treatment or treated with ETV at different times post-infection are shown (red: intracellular HBV DNA, blue: intracellular cccDNA, green: extracellular HBV DNA). The shaded regions correspond to 95% posterior intervals and the solid curves give the best-fit solution (mean) for Eqs (1–4) to the time-course dataset. Error bar represent standard deviation of the mean for three independent samples. All data were fitted simultaneously.

**Fig 2:**
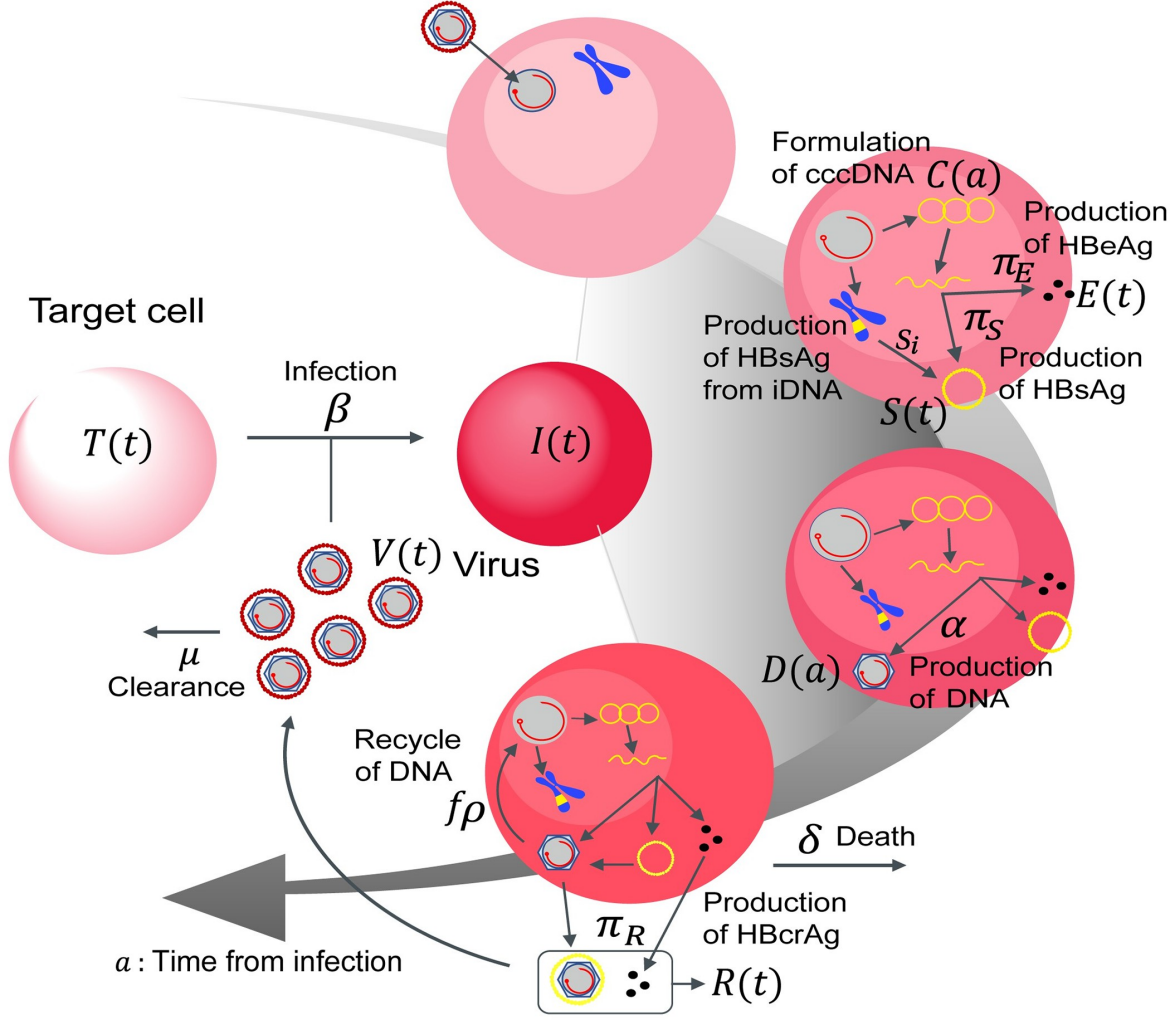
Schematic diagram of multiscale modeling of intracellular and intercellular infection. The entry virion forms cccDNA in the nucleus and produces intracellular HBV DNA at rate *α*. HBsAg, HBeAg, and HBcrAg antigens are also produced from cccDNA at rates *π*_*S*_, *π*_*E*_, and *π*_*R*_ and cleared at *σ* in peripheral blood, respectively. Additionally, HBsAg may also be produced by integrated HBV DNA (iDNA) in the infected cells at a rate *s*_*i*_. The intracellular HBV DNA is consumed at rate *ρ*, of which a fraction 1−*f* of HBV DNA assembled with viral proteins as virus particles are exported from infected cells and the other fraction *f* is reused for further cccDNA formation having a degradation rate of *d*. The infected cells are dead at rate *δ* and the exported viral particles, which are cleared at rate *μ*, infect their target cells at rate *β*.

**Fig 3:**
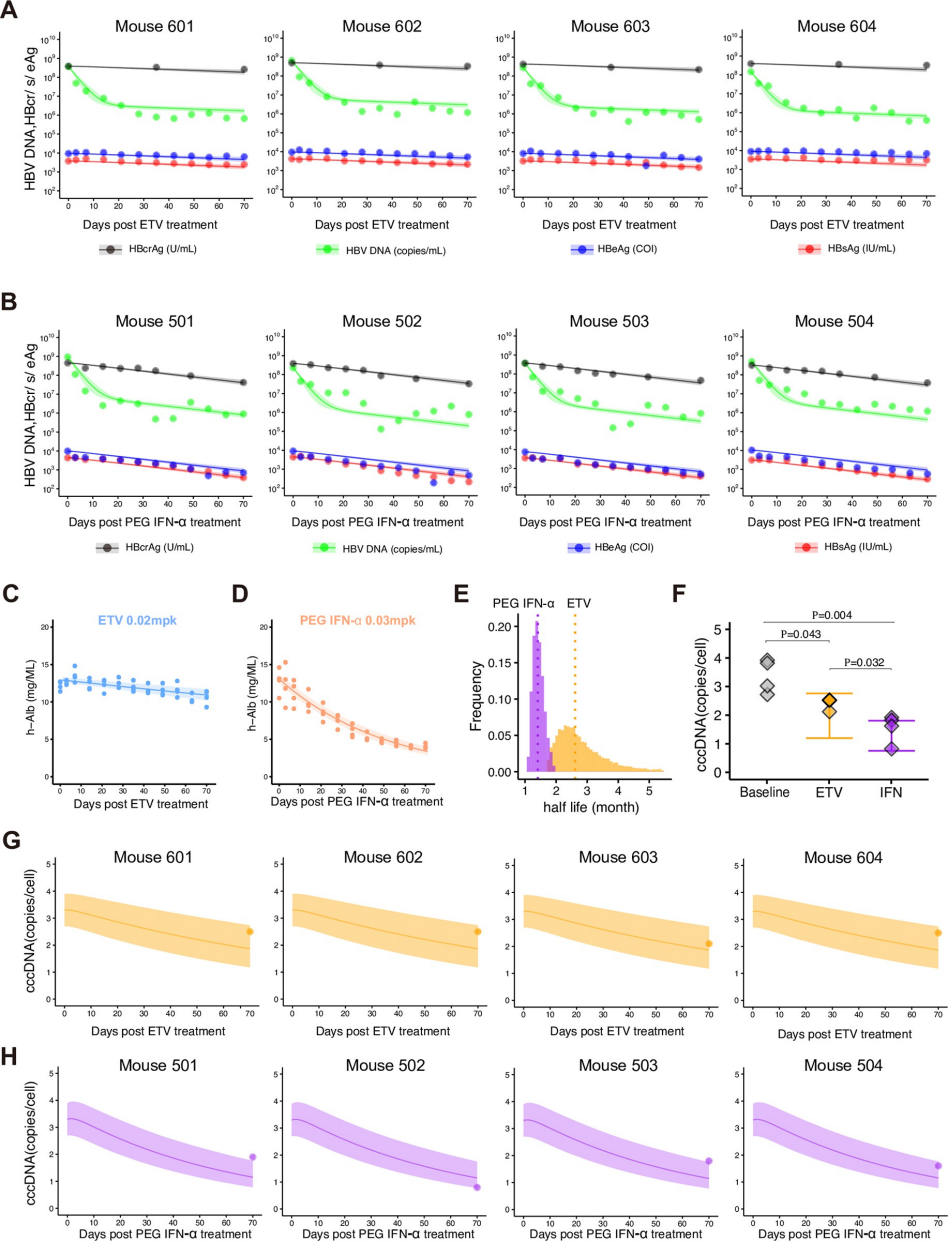
Dynamics of HBV infection in humanized mice. **(A)** and **(B)** show fits of the mathematical model to the extracellular viral markers in peripheral blood of humanized mice treated with ETV or PEG IFN-α (black: HBcrAg, green: HBV DNA, blue: HBeAg, red: HBsAg). The shaded regions correspond to 95% posterior intervals and the solid curves give the best-fit solution (mean) for Eqs (S17-20) or (S30-33) to the time-course dataset. All data were fitted simultaneously. **(C)** and **(D)** show decay characteristics for h-Alb in peripheral blood of humanized mice treated with ETV or PEG IFN-α, respectively. The shaded regions correspond to 95% confidence intervals and the solid curves give the best-fit solution (mean) for a single decay model to the time-course dataset. **(E)** The distribution of the half-life of cccDNA, log 2/*d*, under treatment with PEG IFN-α inferred by MCMC computations. **(F)** Comparisons of predicted cccDNA copies/cell by Eq (S22) or (S35) with estimated parameters and the measured cccDNA levels before (i.e., baseline) and 70 days after ETV or PEG IFN-α treatment in humanized mice. Whiskers show the 95% posterior intervals. **(G)** and **(H)** show that the intrahepatic cccDNA levels (dots) measured in liver samples at 70 days post-treatment with ETV or PEG IFN-α and its model predictions by Eq (S22) or (S35) (lines and shaded regions are the mean and 95% posterior intervals) in orange and purple, respectively.

To predict the amount of intrahepatic cccDNA under antiviral treatment, we developed a multiscale mathematical model that explains the process of intracellular and intercellular HBV propagation using data from cell culture experiments and humanized mice models. First, we used cell culture experiments to create a simple mathematical model of intracellular HBV replication with or without antivirals, which allowed us to measure cccDNA in hepatocytes over time (**Fig 1**). Next, we developed a multiscale model of intracellular and intercellular HBV infection *in vivo* by integrating the simple model into a standard intercellular virus infection dynamics model (**Fig 2**). We used the humanized mice model to evaluate the performance of this multiscale model by measuring longitudinal extracellular viral markers (e.g., HBV DNA, HBsAg, HBcrAg, and HBeAg) in peripheral blood and cccDNA levels in hepatocytes from sacrificed mice before and after treatment (**Fig 3**). We explain our approach in more detail below, highlighting the link between these two models.

### Mathematical model of intracellular HBV replication dynamics: Intracellular data in a cell culture model

To develop a simple mathematical model that reflects the dynamics of HBV propagation including cccDNA, we performed cell culture experiments using PHH because cccDNA can be “directly” quantified in this system (**Figs 1A, A in S1 Text** and **Methods**). PHH were exposed to HBV and PEG8000, which is required to support viral attachment on the cell surface [27], for 16 hours to allow viral entry into the cells. After that, the cells were washed by the addition of fresh medium to remove free HBV and PEG8000, and the cells were then cultured without PEG8000. Thus, the HBV produced by the infected cells does not infect other cells (or the effect of multiple infection by HBV is minor) because PEG8000 was not added to the culture medium after day 1. By applying these experimental conditions, we could exclude viral infectivity from our model. After the initial medium exchange, we replaced the medium every 3–4 days. This method of cell maintenance does not affect the characteristics of PHH, such as the susceptibility to HBV [28]. Note that, these operations reset the released virus, and therefore, it may impact the calculation of the half-life of extracellular HBV DNA. The amounts of extracellular and intracellular HBV DNA and intracellular cccDNA were monitored longitudinally (every 3 to 4 days up to 24–31 days post-inoculation) either with or without ETV treatment (**Figs 1B**, **A in S1 Text** and **Methods**). Note that PHH were maintained at 100% confluent conditions with a 2% concentration of dimethyl sulfoxide (DMSO) in the medium during the entire infection assay to support low cell growth and prevent cell division [29–31].

In the control cells, the level of extracellular HBV DNA increased from day 5 to 20 after a transient decrease (**Fig 1B**). In our experiments, due to the addition of a large amount of input virus as mentioned above, it was not possible to completely remove all of the virus by the wash process 16 hours after infection. Therefore, there is a possibility that the extracellular HBV DNA detected from infection to day 5 may contain input virus. Subsequently, from day 5 to 20, it is believed that the increase in HBV DNA is due to the release of the virus from infected cells into the medium. Infected HBV initially forms cccDNA, followed by the production of intracellular HBV DNA. As a result, the dynamics were observed where cccDNA levels increased earlier than intracellular HBV DNA and reached a plateau. The slope of cccDNA in the first 10 days after infection was estimated by linear regression to be 0.007, indicating little change, followed by a little bit decline or fluctuation. Subsequently, intracellular HBV DNA exhibited a rapid increase from day 1–5, followed by a plateau.

To describe the intracellular virus life cycle in HBV-infected PHH, we developed the following mathematical model (**Fig 1A**):

$$\frac{dC(t)}{dt}=f\rho D(t)-dC(t),$$ (1)

$$\frac{dD(t)}{dt}=\alpha C(t)-\rho D(t),$$ (2)

$$\frac{dQ(t)}{dt}=(1-f)\rho D(t)-d_{E}Q(t).$$ (3)

The variables *C*(*t*), *D*(*t*), and *Q*(*t*) represent the amount of intracellular cccDNA and of intracellular and extracellular HBV DNA in cultures that have been infected for time *t* (later, we consider infection age as *a* instead of *t*), respectively. The intracellular HBV DNA is produced from cccDNA at rate *α* and is lost at rate *ρ*, of which a fraction 1−*f* of HBV DNA is assembled with viral proteins as virus particles that are exported out of infected cells, and the other fraction *f* is reused for further cccDNA formation. The viral particles have a clearance rate *d*_*E*_ due to media replenishment [11,32] and cccDNA has a degradation rate of *d*. We have ignored the degradation of intracellular DNA since it is small compared with the consumption rate of HBV DNA due to virion production [33,34] (see **Table 1**). This intracellular HBV replication model can be modified to include the antiviral effects of different classes of drugs. For example, under treatment with entecavir (ETV), which is a reverse transcriptase inhibitor, the antiviral effect of ETV is assumed to be in blocking HBV DNA production with an effectiveness, *ε*, 0<*ε*≤1, and is modeled by assuming

$$\frac{dD(t)}{dt}=(1-\varepsilon )\alpha C(t)-\rho D(t).$$ (4)

Then we fitted the model to the time-course quantification datasets obtained with and without treatment with ETV (**Methods**). The inhibition of HBV DNA production by ETV perturbates intracellular HBV replication, which enabled us to estimate parameters in the mathematical model [9]. In fact, the amounts of intracellular and extracellular HBV DNA were decreased after treatment with ETV (the middle and right panels in **Fig 1B**) compared with the control experiment (the left panel in **Fig 1B**), whereas the amount of intracellular cccDNA was not changed because of the different time scales of decay for HBV DNA and cccDNA (see **Table 1**). The typical behaviour of the model using these best-fit parameter estimates is shown together with the data in **Fig 1B**, and the estimated parameters and initial values are listed in **Table 1**. Note that, in our mathematical model, we do not account for the "entry process," which means we cannot explicitly incorporate our experimental condition, such as *D*(0) = *C*(0) = 1 and *Q*(0) = 10^8^. Instead, we aim to replicate the high levels of intracellular DNA and cccDNA resulting from the high inoculation dose using these initial values, *D*(0) and *C*(0), respectively.

**Table 1:** Estimated parameters and initial values for HBV infection in PHH.

Parameter or variable	Symbol	Unit	Mean	95% CI
Production rate of HBV DNA from cccDNA	*α*	day^-1^	2.14×10^2^	(0.62−6.32)×10^2^
Fraction of HBV DNA recycling for cccDNA	*f*	---	1.26×10^−5^	2.71×10^−10^−1.38×10^−4^
Degradation rate of cccDNA	*d*	day^-1^	1.90×10^−2^	(0.34−4.58)×10^−2^
Consumption rate of HBV DNA for virion	*ρ*	day^-1^	6.49×10^−1^	0.21−1.77
Clearance rate of extracellular HBV DNA	*d*_*E*_	day^-1^	1.10	0.45−2.47
Inhibition rate of ETV	*ε*	---	0.89	0.75−0.97
Initial value for cccDNA *	*C*(0)	copies/well	2.87×10^4^	(1.38−5.23)×10^4^
Initial value for cccDNA **	*C*(0)	copies/well	2.31×10^4^	(1.21−4.03)×10^4^
Initial value for cccDNA ***	*C*(0)	copies/well	2.36×10^4^	(1.25−403)×10^4^
Initial value for intracellular HBV DNA*	*D*(0)	copies/well	1.89×10^5^	(1.75−7.90)×10^5^
Initial value for intracellular HBV DNA **	*D*(0)	copies/well	3.52×10^5^	(0.03−1.46)×10^5^
Initial value for intracellular HBV DNA ***	*D*(0)	copies/well	1.76×10^5^	(1.80−7.58)×10^5^
Initial value for extracellular HBV DNA *	*Q*(0)	copies/well	1.53×10^8^	1.61×10^4^−1.35×10^9^
Initial value for extracellular HBV DNA **	*Q*(0)	copies/well	3.63×10^8^	2.88×10^4^−3.82×10^9^
Initial value for extracellular HBV DNA ***	*Q*(0)	copies/well	1.80×10^8^	1.66×10^4^−1.62×10^9^

* These values are estimated for condition 1: [No ETV treatment]].

** These values are estimated for condition 2: [ETV treatment from day 1].

*** These values are estimated for condition 3: [ETV treatment from day 10].

It was estimated that 214 copies of HBV DNA are produced from cccDNA in a cell per day on average; only 0.00126% of the produced HBV DNA is recycled to cccDNA. The mean half-life of cccDNA is 51 days in PHH, which is consistent with previous results showing the cccDNA half-life and the limited recycling activity in PHH or patients or HepG2 cells clone expressing NTCP [30,31,35]. We did not consider the degradation of HBV DNA because, although we cannot independently estimate the degradation of HBV DNA, our estimates and conclusions remain consistent due to the minimal proportion of HBV DNA recycled to cccDNA when a fixed value is added to the model. Moreover, this assumption could be supported by a low degradation rate of intracellular HBV DNA [33]. Note that, for more precise parameter estimation, especially regarding the fraction of HBV DNA recycling, it is necessary to measure other variables over time, including the recycling rate of HBV DNA for cccDNA production by viral replication assay using large HBs (L-HBs)-deficient HBV [31]. In cells expressing L-HBs-deficient HBV, because L-HBs is required for the production of infectious virion and viral release, *de novo* infection via produced virions from infected cells does not occur. In other words, the cccDNA produced in these cells is considered to be derived from recycled HBV DNA. Thus, estimation of the recycling ratio could be improved by quantifying the levels of HBV DNA and cccDNA within both wild-type HBV-infected cells and cells infected with L-HBs-deficient HBV. In addition, in the context of modeling the accumulation of cccDNA from day 0 to a designated "day 1," characterized by rapid cccDNA recycling, this parameter can be adjusted post-"day 1" to enhance the precision of our model. Incorporating these additional datasets and assumptions of model may lead to a reduction in the confidence interval of our estimation.

### Multiscale mathematical model of intracellular and intercellular HBV infection dynamics

While we can “directly” monitor cccDNA dynamics in hepatocyte cell culture experiments (**Fig 1B**), it is difficult to obtain time-course measurements of cccDNA *in vivo*. Thus, we next extended the above combined experimental-theoretical approach to describe HBV dynamics *in vivo* and to estimate the cccDNA half-life using extracellular viral markers present in peripheral blood. Here, to precisely quantify both intracellular and intercellular virus dynamics from these viral markers, we derived a multiscale model using PDEs that couple intra-, inter-, and extracellular virus dynamics to analyze multiscale experimental data on HBV infection as follows (c.f. [10]) (**Fig 2**).

Our multiscale model is an extension of the standard mathematical model describing intercellular virus infection dynamics:

$$\frac{dT(t)}{dt}=s-d_{T}T(t)-\beta T(t)V(t),$$

$$\frac{dI(t)}{dt}=\beta T(t)V(t)-\delta I(t),$$

$$\frac{dV(t)}{dt}=pI(t)-\mu V(t).$$

The variables *T*(*t*), *I*(*t*), and *V*(*t*) are the number of uninfected cells, the number of infected cells, and the amount of (extracellular) viruses, respectively. We assumed that uninfected cells are supplied at rate *s*, die at per capita rate *d*_*T*_, and are infected by viruses at rate *β*; that infected cells die at per capita rate *δ*; and that viruses clear at rate *μ* per virion. Since the *de novo* virus production rate *pI*(*t*) is coupled with the intracellular viral replication process, *pI*(*t*) could be replaced with “HBV DNA” that are exported out of infected cells. Given that the amount of viral particles exported from infected cells, i.e., the extracellular HBV DNA load, depends on the time after infection, that is, infection age *a*, we formulate virus production at time *t* as follows:

$$pI(t)=(1-f)\rho \int_{0}^{\infty }D(t,a)i(t,a)da.$$

Here the density of infected cells with infection age *a* is defined as *i*(*t*, *a*), and therefore the total number of infected cells is $I(t)=\int_{0}^{\infty }i(t,a)da$. Consequently, we obtain

$$\frac{dT(t)}{dt}=s-d_{T}T(t)-\beta T(t)V(t),$$ (5)

$$\left(\frac{\partial }{\partial t}+\frac{\partial }{\partial a}\right)i(t,a)=-\delta i(t,a),$$ (6)

$$\frac{dV(t)}{dt}=(1-f)\rho \int_{0}^{\infty }D(t,a)i(t,a)da-\mu V(t),$$ (7)

$$\left(\frac{\partial }{\partial t}+\frac{\partial }{\partial a}\right)C(t,a)=f\rho D(t,a)-dC(t,a),$$ (8)

$$\left(\frac{\partial }{\partial t}+\frac{\partial }{\partial a}\right)D(t,a)=\alpha C(t,a)-\rho D(t,a),$$ (9)

with the boundary condition *i*(*t*, 0) = *βT*(*t*)*V*(*t*) and initial condition *i*(0, *a*) = *i*_0_(*a*). The intracellular variables *C*(*t*, *a*) and *D*(*t*, *a*), which evolve depending on the age *a*, represent the amount of intracellular cccDNA and HBV DNA per infected cell, respectively.

We also defined the following extracellular variables used as “extracellular viral markers” to predict the dynamics of cccDNA in hepatocytes, that is, the amount of HBsAg, HBeAg, and HBcrAg antigens as *S*(*t*), *E*(*t*), and *R*(*t*), respectively:

$$\frac{dS(t)}{dt}=\pi_{S}\int_{0}^{\infty }C(t,a)i(t,a)da+s_{i}\int_{0}^{\infty }i(t,a)da-\sigma S(t),$$ (10)

$$\frac{dE(t)}{dt}=\pi_{E}\int_{0}^{\infty }C(a)i(t,a)da-\sigma E(t),$$ (11)

$$\frac{dR(t)}{dt}=\pi_{R}\int_{0}^{\infty }C(t,a)i(t,a)da-\sigma R(t),$$ (12)

Note that HBsAg, HBeAg, and HBcrAg antigens are produced from cccDNA in infected cells at rates *π*_*S*_, *π*_*E*_, and *π*_*R*_ and are cleared at rate *σ*, respectively. Additionally, HBsAg may also be produced by integrated HBV DNA (iDNA) in the infected cells at a rate *s*_*i*_. The definition of an age-structured population model is found in [36].

### Analyzing extracellular viral markers by use of a humanized mice model

To check the performance of our multiscale model, we conducted an HBV infection experiment with humanized liver urokinase-type plasminogen activator/severe combined immunodeficiency (uPA/SCID mice). The mice have the SCID phenotype, which leads to a deficiency in the function of T cells and B cells [27,37]. Mice maintain their innate immune function, and it is known that the expression of interferon-stimulated genes (ISGs) is induced upon PEG IFN-α treatment [38,39]. Thus, evaluating viral decay with PEG IFN-α using this mouse model should be useful. After the mice were inoculated with HBV and a sustained HBV DNA load was reached (approximately 5.6×10^8^ copies/ml) at 53 days post-inoculation, the mice were treated with or without ETV or PEG IFN-α continuously. We then longitudinally monitored four different viral markers in the peripheral blood every 3 to 7 days up to 70 days post-treatment: extracellular HBV DNA, HBcrAg, HBeAg, and HBsAg (**Fig A in S1 Text** and **Methods**).

Administering ETV to HBV-infected mice induced rapid reduction of extracellular HBV DNA, reaching a plateau thereafter. Since ETV specifically inhibits the reverse transcription of the virus, it had little impact on other circulating virus markers including extracellular HBcrAg, HBeAg, and HBsAg (**Fig 3A**). Similarly, PEG IFN-α rapidly decreased extracellular HBV DNA, and the subsequent dynamics of viral load varied among mice but showed an overall trend of gradual reduction. In contrast to the ETV-administrated group, all three other viral markers continued to decrease during the PEG IFN-α administration period (**Fig 3B**). Since associations between measured cccDNA and serum markers were found, it is necessary to investigate their dynamics with mathematical modeling (**Fig B in S1 Text**). While h-Alb levels remained constant during ETV administration, they continued to decrease with PEG IFN-α administration. Similar observation decreasing h-Alb by PEG IFN-α administration have been observed in previous study, and it might be attributed to apoptosis induced by ISGs produced via PEG IFN-α administration [28].

We used the multiscale mathematical model of HBV infection (Eqs (5–12)), in which an infected cell produces progeny HBVs extracellularly that are then degraded or infect other cells. We derived simple linearized equations (Eqs (S17-S20) and Eqs (S30-S33)) for fitting to the time-course datasets quantified with mice with or without ETV or PEG IFN-α treatment (**Notes G, H, and I in S1 Text**). Here we assumed the proportion of HBsAg produced from iDNA (i.e., *x* in **Notes H and I in S1 Text**) is fixed at 0 (all HBsAg is from cccDNA), 0.5 (HBsAg equally from cccDNA and iDNA), or 0.8 (HBsAg dominantly from iDNA) as our sensitivity analysis. Note that the decay rates of infected cells were estimated separately from human albumin in peripheral blood of humanized mice (**Fig 3C and 3D**) and the clearance rates of extracellular HBV DNA and antigens were fixed as previously estimated values, that is, *μ* = 16.1 d^-1^ [40] and *σ* = 1.00 d^-1^[41,42]. Regardless of the proportion, we showed that the model well captured the experimental quantification data (i.e., extracellular HBV DNA, HBcrAg, HBeAg, and HBsAg) over time with best-fit parameters (**Figs 3A, 3B** and ** in S1 Text**). However, comparing SSR (SSR = 19.9, 23.1, 31.9 for *x* = 0, 0.5, 0.8, respectively), we found that a model considering that cccDNA is the dominant source of HBsAg (i.e., *x* = 0) best described our data. In the following, we discuss the parameter values of the multiscale mathematical model with no HBsAg production from iDNA (see **Discussion**). The estimated parameters and fixed initial values are listed in **Tables 2, D** and **E in S1 Text**, respectively.

**Table 2:** Estimated parameters for HBV infection in humanized mouse.

Parameters or variables	Symbol	Unit	Mean	95% CI
Combined parameter^†^	*fα*	-	4.1×10^−3^	(1.5−8.4)×10^−3^
Inhibition rate of HBV DNA production	*ε*	-	9.6×10^−1^	(9.4−9.8)×10^−1^
Decay rate of infected cells*	δ	day^-1^	2.4×10^−3^	(1.6−3.2)×10^−3^
Decay rate of infected cells with IFN-α*	δ_IFN_	day^-1^	1.9×10^−2^	(1.7−2.1)×10^−2^
Degradation rate of cccDNA	*d*	day^-1^	8.8×10^−3^	(4.2−13)×10^−3^
Degradation rate of cccDNA with IFN-α	*d*_IFN_	day^-1^	1.6×10^−2^	(1.2−2.1)×10^−2^
Release rate of intracellular HBV DNA	*ρ*	day^-1^	3.8×10^−1^	(2.9−5.2)×10^−1^

^†^ Production rate of HBV DNA from cccDNA × Fraction of HBV DNA recycling for cccDNA.

* These values are estimated from h-Alb kinetics.

### Predicting intrahepatic cccDNA dynamics from extracellular viral makers

When we applied the multiscale mathematical model to the evaluation of the drug effects on viral replication and amount of cccDNA, we assumed that ETV almost completely blocks intracellular HBV replication and *de novo* infections (i.e., potent antiviral effect) but has no direct effect on cccDNA degradation, as reported previously (**Note H in S1 Text**) [22,43–45].

We found the mean half-life of cccDNA was 109 days in the humanized mice under ETV treatment (**Fig 3E** and **Table 2**). In addition to the potent antiviral effect of PEG IFN-α (i.e., no *de novo* infections, see **Note I in S1 Text**) as in other reports [46], our analysis demonstrated that PEG IFN-α treatment significantly reduced the half-life of cccDNA to around 49 days (**Fig 3E** and **Table 2**), implying PEG IFN-α promotes cccDNA degradation. This calculation is supported by our previous mouse experiments showing that PEG IFN-α treatment for 42 days reduces cccDNA levels to 23–33%, which was semi-quantified by Southern blot analysis [47] (**Table F in S1 Text**). Note that the estimated cccDNA half-life under antiviral treatment is based on the assumption that no *de novo* infections occur because of the strong antiviral effects (i.e., assuming a perfect inhibition; *β* = 0). The actual cccDNA half-life may be even shorter if low-level de novo infections occur during ETV and/or PEG IFN-α treatment (**Notes H and I in S1 Text**). We address this point further in the **Discussion**.

Importantly, the intrahepatic cccDNA levels were experimentally measured in liver samples from humanized mice. This was done by collecting the liver from sacrificed mice and digesting the tissue with plasmid-safe ATP-dependent deoxyribonuclease DNase (PSAD), followed by absolute quantification using droplet digital PCR (ddPCR) [47,48]. To validate our multiscale modeling, including Eq (S22) and Eq (S35) in **Notes H and I in S1 Text**, as well as the estimated parameters listed in **Table 2**, we conducted model simulations using accepted Markov chain Monte Carlo (MCMC) parameter estimates (**Fig 3A and 3B** and **Methods**). Unfortunately, we cannot measure the cccDNA level before treatment without sacrificing the mice. Therefore, in our simulation, to address the variability in initial cccDNA levels, we sampled 100 initial values within the range defined by the minimum and maximum cccDNA levels observed in non-treated humanized mice (i.e., the “baseline” in **Fig 3F**). We then used this set of initial cccDNA values for simulating cccDNA dynamics.

While we acknowledge that there is a small discrepancy between our simulation and measurements on the cccDNA level, the simulations confirmed that the cccDNA levels were roughly within the simulated range depicted in **Fig 3F**. They may also capture the dynamics of cccDNA during the 70 days of ETV and PEG IFN-α treatments as depicted in **Fig 3G** and 3**H**, respectively, as follows. The cccDNA levels in HBV-infected mice decreased gradually with ETV treatment, reaching an average of 2.4 copies/cell (with a maximum of 2.5 copies/cell and a minimum of 2.1 copies/cell) on day 70 after drug administration (**Fig 3G**). On the other hand, PEG IFN-α treatment led to a more rapid reduction in cccDNA levels, with an average of 1.5 copies/cell (maximum 1.9 copies/cell, minimum 0.8 copies/cell) on day 70 (**Fig 3H**). Since cccDNA levels of these mice were normalized based on the cell number measured by the hRPP30 copy number variation assay [49], the variability in cccDNA is presumed to be due to differences in the effectiveness of the drugs in each mouse. The variance of cccDNA level was higher with PEG IFN-α treatment (0.29) than in ETV treatment (0.04). Our assumption regarding the initial values may result in imperfect predictions of cccDNA levels due to individual heterogeneity in baseline cccDNA levels. We further discuss this limitation in **Discussion** section.

## Discussion

So far, mathematical models with several “compartmentalized stages” of intracellular HBV replication (e.g., models described by ODEs which cannot explain time-dependent extracellular viral marker production) have been proposed [12,13]. Here, we developed a multiscale mathematical model that explicitly includes intracellular and intercellular HBV infection, described by age-structured PDEs, for quantifying HBV viral dynamics using in vitro and *in vivo* experimental data. We then predicted the amount of intrahepatic cccDNA using specific viral markers in serum samples (i.e., HBV DNA, HBsAg, HBeAg, and HBcrAg). Our mathematical model using the results from the humanized mouse model without HBsAg produced from iDNA was well fitted. In other words, the levels of iDNA-derived HBsAg are assumed to be negligible compared with those derived from cccDNA. This is because cccDNA is the major source of HBsAg in HBeAg-positive patients and animal models [50]. Our results are further supported by a previous paper, which suggested that iDNA contributes minorly to HBsAg production in the mouse model [51]. On the other hand, other studies have reported that, rather than cccDNA, iDNA of the HBsAg region may contribute to HBsAg production in HBeAg-negative patients [5,6,50]. When we apply our multiscale model to quantify intrahepatic cccDNA in HBV-infected individuals, it will be necessary to include HBsAg produced from iDNA, especially for HBeAg-negative patients. Furthermore, detection of HBeAg in patients may serve as a crucial indicator, as it may differ in response to HBV decay and clearance during antiviral treatment [52]. Therefore, it is important to analyze these groups separately when predicting the antiviral effect of IFN. Some HBeAg-positive cases may undergo seroconversion to HBeAg-negative as treatment progresses. In such cases, it has been reported that the efficacy of the antiviral effect of interferon may decrease [53,54]. Therefore, the HBeAg status of patients is also crucial in predicting the antiviral effects of IFN, and developing mathematical models that take this into account is highly effective for forecasting HBV treatment outcomes.

In this study, we calculated cccDNA copy number and half-life in PHH and humanized mice. Concerning copy number, the number of cccDNA copies was higher in PHH than in mouse. Recent studies have reported little amplification of cccDNA copy number in infected cells after primary infection [55]. This suggests that cccDNA copies in infected cells depend on the amount of cccDNA formed during the initial infection, which correlates with the higher amount of cccDNA in PHH, which are exposed to a large amount of HBV during the initial infection, and the lower amount in humanized mice [56,57]. On the other hand, cccDNA half-life was shorter in PHH than in humanized mouse. cccDNA persistence in hepatocytes requires supplementation of cccDNA by intracellular recycling of the viral genome and/or de novo infection [55]. Although viral recycling will occur in PHH and humanized mice, de novo reinfection is rarely observed in PHH. This is because viral infection in PHH requires the addition of PEG8000 [55], which was not added after the initial infection in this experiment. Thus, one of the pathways for maintaining cccDNA levels does not work in PHH, resulting in a calculated cccDNA half-life that is shorter than that in humanized mice.

Our study has some limitations as follows. The first concerns the experimental quantification method of cccDNA. We quantified cccDNA by PCR-based methods because of the requirement for a large number of quantifications for the mathematical model. Standardization of the detection method for cccDNA by real-time PCR has been discussed over the years [47,48]. We have to be careful about possible overestimation of the amount of cccDNA, even if we minimize the contamination of rcDNA by PSAD digestion as in this study. However, the cccDNA half-life value estimated by our method is roughly unaffected by a slight shift of cccDNA levels. We minimized this limitation by comparing the PCR-based cccDNA quantification data with the values detected by Southern blot in HBV-infected chimeric mice (**Fig 3F, Tables 2,** and **F in S1 Text**). Second, our mathematical model has a few assumptions underlying intracellular and intercellular HBV propagation. We assumed negligible *de novo* infections under ETV and PEG IFN-α treatment because NAs and PEG IFN-α inhibit HBV replication by around 100% (**Notes H and I in S1 Text**). The assumption may overestimate the mean half-life of cccDNA. After additional datasets on the time-course of the viral markers with different intensities of NAs and PEG IFN-α treatments become available, we can determine the inhibition rate more precisely, and our estimation will be improved. Another limitation in our approach is the necessity of the initial value of cccDNA to predict its dynamics. As discussed, in the humanized mouse model, we cannot measure the cccDNA in the liver without sacrificing the mice. This indicates that we cannot measure the cccDNA level more than once in the same individual; that is, the cccDNA level either before or after treatment could be obtained. An interesting possibility of our approach is clinical datasets obtained from HBV patients who were treated with NAs and PEG IFN-α with paired liver biopsies to monitor the cccDNA levels in the liver. Although the current simple but quantitative mathematical model roughly predicted the amount of cccDNA in humanized mice from our extracellular viral markers, more detailed mathematical modeling and complete datasets that address these limitations will be beneficial for a more precise estimation of cccDNA dynamics.

In summary, our multiscale mathematical model combined with the extracellular viral markers, i.e., HBsAg, HBcrAg, HBeAg, and HBV DNA, predicts the amount of intrahepatic cccDNA in vivo and may open new avenues for understanding cccDNA dynamics in patients.

## Methods

### Ethics statement

HBV infection assay using mouse model was performed at Phoenix Bio Co., Ltd. (Hiroshima, Japan). Ethical approval for this study was granted by: the Animal Welfare Committee of PhoenixBio Co., Ltd. (registration number 2200). All efforts were made during the study to minimize animal suffering and to reduce the number of animals used in the experiments.

### HBV infection in primary human hepatocytes

PHH used for the HBV infection assay were maintained according to the manufacturer’s protocol (Phoenix Bio Co., Ltd, Hiroshima, Japan). HBV (genotypeD) used as the inoculum was recovered from the culture supernatant of Hep38.7-Tet cells cultured under tetracycline depletion and concentrated up to 200-fold by polyethylene glycol concentration [58]. PHH were seeded into 96-well plates at 7×10^4^ cells/well and were inoculated with HBV at 8,000 genome equivalents (GEq)/cell in the presence of 4% polyethylene glycol 8,000 (PEG8000) for 16 h. After washing out free HBV, we replenished it with fresh medium every 3 to 4 days. To exclude the possibility that contamination of input virus, we repeated the wash prosses at the time of medium exchange every 3 to 4 days. PHH were continuously treated with ETV at 1 μM or were not treated (control). Cell division is known to reduce the cccDNA per cell in HBV-infected cells [30]; therefore, to avoid this, we maintained PHH at 100% confluent conditions during the entire infection assay. Moreover, a high concentration of DMSO was included in the culture medium as described previously [28], which does not allow cell growth and prevents cccDNA loss by cell division [29–31]. Since we had confirmed by cell counting that human hepatocytes did not significantly proliferate over one month under the above primary culture conditions [28], cell growth dynamics were ignored in our analysis. Culture supernatant was collected from HBV-infected cells and the cells were recovered to quantify HBV DNA in the culture supernatant, total HBV DNA in the cells, and cccDNA by real-time PCR. For real-time PCR, the primer-probe sets used in this study were 5’-AAGGTAGGAGCTGAGCATTCG-3’, 5’-AGGCGGATTTGCTGGCAAAG-3’, and 5’-FAM-AGCCCTCAGGCTCAGGGCATAC-TAMRA-3’ for detecting HBV DNA and 5’-CGTCTGTGCCTTCTCATCTGC-3’, 5’-GCACAGCTTGGAGGCTTGAA-3’, and 5’-CTGTAGGCATAAATTGGT(MGB)-3’ for cccDNA [58].

In the assay shown in **Fig 1**, a large amount of HBV (8000 GEq/cell) is exposed to PHH on day 0, which is the condition in which about 80% of the PHH is infected as reported previously [28]; thus, the cccDNA amount is high at day 1. Previous papers also reported that cccDNA is readily detected as early as day 2 after HBV inoculation and remains at a similar level over time [31,59]. Note that the time “day 1” in our study means 24 h after the end of HBV inoculation (16 h), indicating 40 h after starting HBV inoculation. In addition, HBV infection did not spread because PEG8000, which supports viral attachment on the cell surface [27], was not added to the culture medium after day 1, which resulted in the cccDNA initially forming in the cells without increasing, showing similar amounts of cccDNA on day 1 and day 31.

### HBV infection of humanized mouse

Humanized mice were purchased from Phoenix Bio Co., Ltd. (Hiroshima, Japan). The animal protocol was approved by the Ethics Committees of Phoenix Bio Co., Ltd (Permit Number:2200). These mice were infected with HBV at 1.0×10^6^ copies/mouse that was obtained from human hepatocyte chimeric mice previously infected with genotype C2/Ce, as described previously [60]. Day 53 after inoculation, HBV-infected mice, which showed a plateau of HBV levels in serum, were treated with ETV (at a dose of 0.02 mg/kg, once a day) or PEG IFN-α (at a dose of 0.03 mg/kg, twice a week) continuously for over 70 days (**Figs 3A and 3B** and **A in S1 Text**). The human albumin level in the serum was measured as described previously [37]. The HBV DNA titer was measured by real-time PCR as previously described [61]. HBsAg, HBcrAg, and HBeAg were measured by chemiluminescent enzyme immunoassay using a commercial assay kit (Fujirebio Inc., Tokyo, Japan). The detection limit of the HBsAg assay and HBcrAg assay were 0.005 IU/ml and 1.0 kU/ml, respectively. The cut-off index (COI) of the HBeAg was <1.00 (**Figs 3A and 3B** and **C in S1 Text**). Intrahepatic HBV cccDNA was extracted from a dissected liver treated with PSAD to digest genomic DNA and rcDNA as described previously [62] (**Fig 3F**). Genomic DNA was isolated from the livers of chimeric mice using the phenol/chloroform method as previously described [63]. The cccDNA-specific primer-probe set for cccDNA amplification was used for ddPCR assay [62]. After the generation of reaction droplets, intrahepatic cccDNA was amplified using a Thermal Cycler (Bio-Rad, Hercules, California, USA). In all cases, intrahepatic cccDNA values were normalized by the cell number measured by the hRPP30 copy number variation assay (Bio-Rad, Pleasanton, California, USA) [49]. Of note, hRPP30 levels were separately determined using DNA that was not treated with PSAD.

A previous report using humanized mice [40] showed that serum HBV DNA 10^9^ copies/mL of the mice infected with the same HBV strain was in steady state, indicating an extended phase of continuous HBV expansion. Note that immunostaining for HBcAg in the livers during steady state revealed that most hepatocytes were HBV positive, suggesting that the human hepatocytes of the chimeric mice in this study were sufficiently infected with HBV. In this study, chimeric mice with more than 90% replacement of human hepatocytes were used in the dosing test with PEG IFN or NAs. After HBV inoculation, the serum HBV DNA levels before dosing reached a plateau on day 53 (approximately 5.6×10^8^ copies/mL), suggesting that most human hepatocytes are sufficiently infected with HBV.

### Data fitting and parameter estimation

#### (1) Data analysis for HBV infection on PHH

We categorized datasets as follows: [condition 1 = No ETV treatment], [condition 2 = ETV treatment from day 1], and [condition 3 = ETV treatment from day 10] (**Fig A in S1 Text**). To assess the variability of kinetic parameters and model predictions, we performed Bayesian inference for the dataset of conditions 1, 2, and 3 by using MCMC sampling [11]. A statistical model adopted from Bayesian inference assumed that measurement error followed a normal distribution with mean of zero and constant variance (error variance). Simultaneously, we fitted Eqs (1–3) and Eqs (1, 2 and 4) to the experimental data of intracellular HBV DNA and cccDNA, and extracellular HBV DNA in condition 1 and conditions 2, 3, respectively (**Fig 1B**). Note that we estimated model parameters (i.e., *α*, *f*, *d*, *ρ*, *d*_*E*_, *ε*) for all conditions as common values because the HBV used in this assay was identical. On the other hand, susceptibility and permissiveness of PHH to HBV are known to be heterogeneous; thus, we used different initial values (i.e., *C*(0), *D*(0), *Q*(0)) for each condition (**Table 1**). Distributions of model parameters and initial values were inferred directly by MCMC computations [11].

To assess the identifiability of parameters, we examined parameter collinearity. Collinearity is a measure of the degree of linear interdependence among the parameters. To carry out this analysis, we utilized the FME package [64]. Our model’s collinearity index was 3.4, which meets the criterion for identifiability, as it falls well below the threshold of 20 [33].

#### (2) Data analysis for HBV infection on humanized mouse

To quantify HBV infection and the antiviral effect of ETV or IFN-α in humanized mice, we also performed Bayesian inference using MCMC sampling (10000 among 100000 chains were used as burn-in) because the inter-individual variations are almost negligible. The 95% posterior interval and corresponding cccDNA predictions in each panel of **Fig 3A and 3B** are produced from 100 randomly chosen inferred parameter sets [64]. We here used a previously estimated half-life of extracellular HBV DNA in peripheral blood, that is, 62 minutes (*μ* = 16.1 d^-1^) [40], and that of extracellular HBsAg in peripheral blood, 0.69 day (*σ* = 1 d^-1^) [41,42]. Simultaneously, we fitted Eqs (*S*17-*S*20) and Eqs (*S30*-*S*33) to the experimentally measured extracellular HBV DNA, HBcrAg, HBeAg, and HBsAg obtained from HBV-infected humanized mice treated with ETV and PEG IFN-α, respectively (**Figs 3A, 3B** and **3C in S1 Text**), and estimated *d*, *d*_*IFN*_ and *ρ* (**Tables 2** and **D in S1 Text**). Note that we fixed all initial values as initial points of our dataset (**Table E in S1 Text**), and the decay rates of infected cells were separately estimated from h-Alb in peripheral blood of the humanized mice (**Fig 3C and 3D**, **Tables 2** and **E in S1 Text**).

### Statistical analysis

Mathematical modeling, transformation to the reduced model, and its linearization are described in **Notes G, H and I in S1 Text** in detail. All analyses of samples were conducted using custom scripts in R and were visualized using RStudio. For comparisons between groups, Mann-Whitney U tests and t-test were used. All tests were declared significant for *p*<0.05.

## Supporting information

S1 Text Fig A. Summary of HBV infection datasets. Fig B. Correlation between the biomarkers and cccDNA. Fig C. Fitting of the mathematical model to the extracellular viral markers in peripheral blood of humanized mice treated with ETV or PEG IFN-α considering HBsAg production from iDNA. Table D. Estimated parameters for HBV infection in humanized mouse considering HBsAg production from iDNA. Table E. Fixed initial values for HBV infection in humanized mouse. Table F. Quantified results for cccDNA in HBV-infected mouse. Note G. Transformation to a system of ODEs from a PDE multiscale model. Note H. Linearized equations under potent NAs treatment in humanized mouse. Note I. Linearized equations under potent PEG IFN-α treatment in humanized mouse.
(DOCX)
