# Supplementary material for: Multiscale modeling of HBV infection integrating intra- and intercellular viral propagation to analyze extracellular viral markers
Source: PLoS Comput Biol. 2024 Mar 11;20(3):e1011238. doi: 10.1371/journal.pcbi.1011238 (PMC10957078; doi:10.1371/journal.pcbi.1011238)
Supplement: S1 Text — Fig A. Summary of HBV infection datasets. Fig B. Correlation between the biomarkers and cccDNA. Fig C. Fitting of the mathematical model to the extracellular viral markers in peripheral blood of humanized mice treated with ETV or PEG IFN-α considering HBsAg production from iDNA. Table D. Estimated parameters for HBV infection in humanized mouse considering HBsAg production from iDNA. Table E. Fixed initial values for HBV infection in humanized mouse. Table F. Quantified results for cccDNA in HBV-infected mouse. Note G. Transformation to a system of ODEs from a PDE multiscale model. Note H. Linearized equations under potent NAs treatment in humanized mouse. Note I. Linearized equations under potent PEG IFN-α treatment in humanized mouse. (DOCX) [file pcbi.1011238.s001.docx]

**Supplementary Information**

Multiscale modeling of HBV infection integrating intra- and intercellular viral propagation to analyze extracellular viral markers

Kosaku Kitagawa^1,†^, Kwang Su Kim^1,2,†^, Masashi Iwamoto^1,3,‡^, Sanae Hayashi^4,‡^, Hyeongki Park^1,‡^, Takara Nishiyama^1^, Naotoshi Nakamura^1^, Yasuhisa Fujita^1^, Shinji Nakaoka^5^, Kazuyuki Aihara^6^, Alan S. Perelson^7^, Lena Allweiss^8,9^, Maura Dandri^8,9^, Koichi Watashi^3,10,11,#,*^, Yasuhito Tanaka^4^ and Shingo Iwami^1,11,12,13,14,15,#,*^

^1^interdisciplinary Biology Laboratory (iBLab), Division of Natural Science, Graduate School of Science, Nagoya University; Nagoya, Japan. ^2^Department of Scientific Computing, Pukyong National University; Busan, South Korea. ^3^Department of Virology II, National Institute of Infectious Diseases; Tokyo, Japan. ^4^Department of Gastroenterology and Hepatology, Faculty of Life Sciences, Kumamoto University; Kumamoto, Japan. ^5^Faculty of Advanced Life Science, Hokkaido University; Sapporo, Japan. ^6^International Research Center for Neurointelligence, The University of Tokyo Institutes for Advanced Study, The University of Tokyo; Tokyo, Japan. ^7^Theoretical Biology and Biophysics Group, Los Alamos National Laboratory; Los Alamos, USA. ^8^Department of Internal Medicine, University Medical Center Hamburg-Eppendorf; Hamburg, Germany. ^9^German Center for Infection Research (DZIF), Hamburg-Lübeck-Borstel-Riems partner sites; Germany. ^10^Research Center for Drug and Vaccine Development, National Institute of Infectious Diseases; Tokyo, Japan. ^11^Department of Applied Biological Sciences, Faculty of Science and Technology, Tokyo University of Sciences; Chiba, Japan. ^12^Institute of Mathematics for Industry, Kyushu University; Fukuoka, Japan. ^13^Institute for the Advanced Study of Human Biology (ASHBi), Kyoto University; Kyoto, Japan. ^14^NEXT-Ganken Program, Japanese Foundation for Cancer Research (JFCR); Tokyo, Japan. ^13^Interdisciplinary Theoretical and Mathematical Sciences (iTHEMS), RIKEN; Wako, Japan. ^15^Science Groove Inc.; Fukuoka, Japan.

**
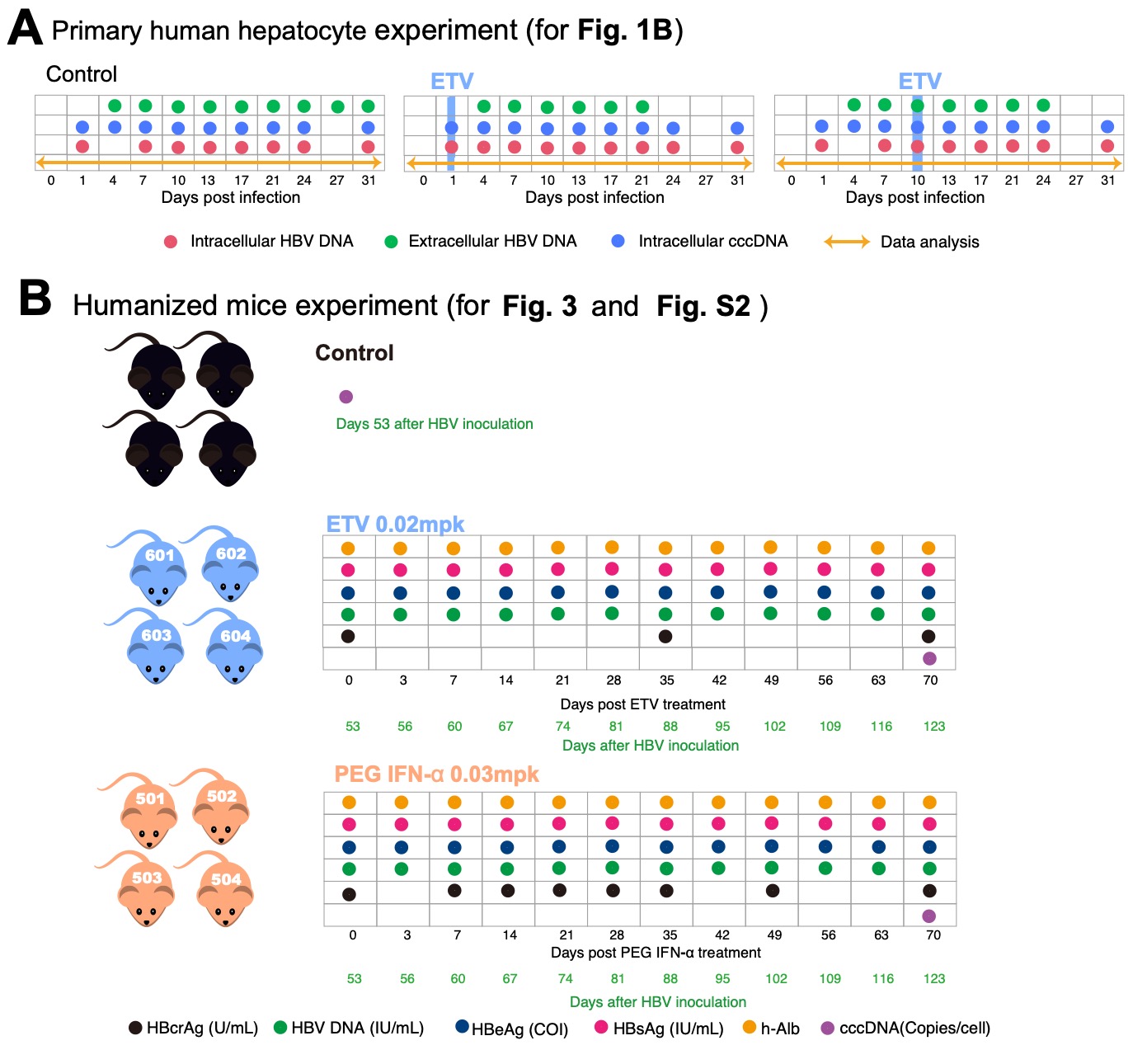
Fig A. Summary of** **HBV infection datasets:** Detailed data-sampling schedule for HBV-infected **(A)** primary human hepatocytes, and **(B)** humanized mice. We used an open source(https://openclipart.org/) for the clipart.


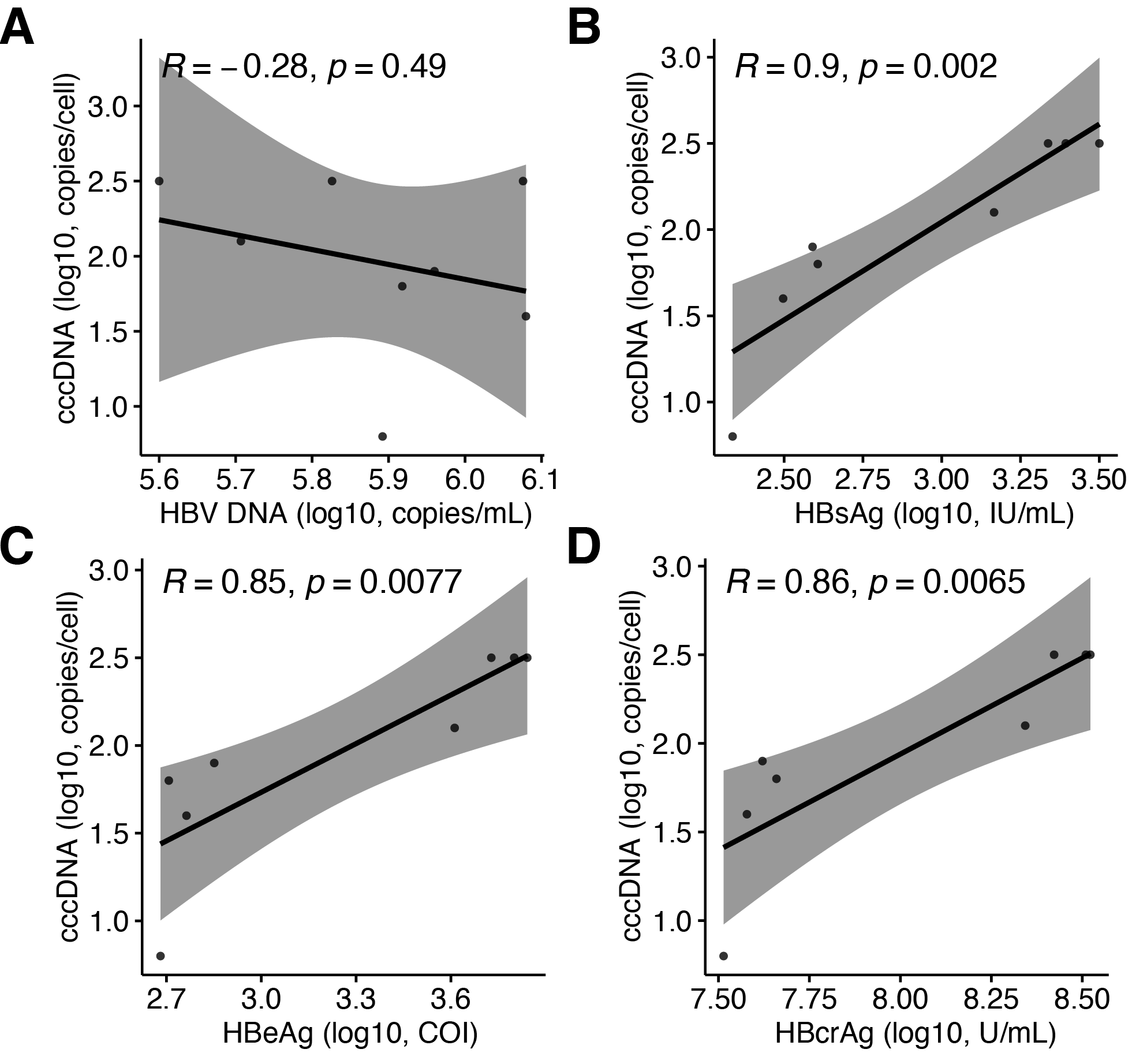


**Fig B. Correlation between the biomarkers and cccDNA:** The relationship between the measured (A) HBV DNA, (B) HBsAg, (C) HBeAg and (D) HBcrAg and the cccDNA on last measurement day, are shown, respectively. The R and p on the top of each panel indicate the Pearson’s correlation and its p-value, respectively.

**Fig C. Dynamics of viral makers in HBV infected humanized mice:** Fitting of the mathematical model to the extracellular viral markers in peripheral blood of humanized mice treated with ETV or PEG IFN-α considering HBsAg production from iDNA ($x=0.5$ or $0.8$).

**Table D. Estimated parameters for HBV infection in humanized mouse considering HBsAg production from iDNA**

| **Parameters or variables** | **Symbol** | **Unit** | **Value** |
| --- | --- | --- | --- |
| $\boldsymbol{x=0.5}$ |  |  |  |
| Combined parameter^†^ | $f\alpha$ | - | $5.4\times{10}^{-3}$ |
| Inhibition rate of HBV DNA production | $\varepsilon$ | - | $9.7\times{10}^{-1}$ |
| Decay rate of infected cells | $\delta$ | day^-1^ | $2.4\times{10}^{-3}$ |
| Decay rate of infected cells with IFN-α | $\delta_{\mathrm{IFN}}$ | day^-1^ | $1.9\times{10}^{-2}$ |
| Degradation rate of cccDNA | $d$ | day^-1^ | $1.2\times{10}^{-2}$ |
| Degradation rate of cccDNA with IFN-α | $d_{\mathrm{IFN}}$ | day^-1^ | $1.7\times{10}^{-2}$ |
| Release rate of intracellular HBV DNA | $\rho$ | day^-1^ | $3.8\times{10}^{-1}$ |
| Residual sum of squares | -- | - | $23.123$ |
| $\boldsymbol{x=0.8}$ |  |  |  |
| Combined parameter^†^ | $f\alpha$ | - | $1.3\times{10}^{-2}$ |
| Inhibition rate of HBV DNA production | $\varepsilon$ | - | $9.9\times{10}^{-1}$ |
| Decay rate of infected cells | $\delta$ | day^-1^ | $2.4\times{10}^{-3}$ |
| Decay rate of infected cells with IFN-α | $\delta_{\mathrm{IFN}}$ | day^-1^ | $1.9\times{10}^{-2}$ |
| Degradation rate of cccDNA | $d$ | day^-1^ | $4.6\times{10}^{-4}$ |
| Degradation rate of cccDNA with IFN-α | $d_{\mathrm{IFN}}$ | day^-1^ | $1.2\times{10}^{-3}$ |
| Release rate of intracellular HBV DNA | $\rho$ | day^-1^ | $3.2\times{10}^{-1}$ |
| Residual sum of squares | -- | - | $31.947$ |

^†^ Production rate of HBV DNA from cccDNA $\times$ Fraction of HBV DNA recycling for cccDNA

**Table E. Fixed initial values** **for HBV infection in humanized mouse**

| **Variable** | **Symbol** | **Unit** | **Value** |
| --- | --- | --- | --- |
| **ETV** |  |  |  |
| Initial value for extracellular HBV DNA for Mouse 601 | $V\left( 0 \right)$ | copies/ml | $3.68\times{10}^{9}$ |
| Initial value for extracellular HBsAg for Mouse 601 | $S\left( 0 \right)$ | IU/ml | $3.75\times{10}^{3}$ |
| Initial value for extracellular HBeAg for Mouse 601 | $E\left( 0 \right)$ | COI | $9.41\times{10}^{3}$ |
| Initial value for extracellular HBcrAg for Mouse 601 | $R\left( 0 \right)$ | U/ml | $3.85\times{10}^{9}$ |
| Initial value for extracellular HBV DNA for Mouse 602 | $V\left( 0 \right)$ | copies/ml | $6.53\times{10}^{9}$ |
| Initial value for extracellular HBsAg for Mouse 602 | $S\left( 0 \right)$ | IU/ml | $4.14\times{10}^{3}$ |
| Initial value for extracellular HBeAg for Mouse 602 | $E\left( 0 \right)$ | COI | $9.52\times{10}^{3}$ |
| Initial value for extracellular HBcrAg for Mouse 602 | $R\left( 0 \right)$ | U/ml | $4.97\times{10}^{9}$ |
| Initial value for extracellular HBV DNA for Mouse 603 | $V\left( 0 \right)$ | copies/ml | $2.82\times{10}^{9}$ |
| Initial value for extracellular HBsAg for Mouse 603 | $S\left( 0 \right)$ | IU/ml | $3.22\times{10}^{3}$ |
| Initial value for extracellular HBeAg for Mouse 603 | $E\left( 0 \right)$ | COI | $8.13\times{10}^{3}$ |
| Initial value for extracellular HBcrAg for Mouse 603 | $R\left( 0 \right)$ | U/ml | $4.25\times{10}^{9}$ |
| Initial value for extracellular HBV DNA for Mouse 604 | $V\left( 0 \right)$ | copies/ml | $1.48\times{10}^{9}$ |
| Initial value for extracellular HBsAg for Mouse 604 | $S\left( 0 \right)$ | IU/ml | $3.56\times{10}^{3}$ |
| Initial value for extracellular HBeAg for Mouse 604 | $E\left( 0 \right)$ | COI | $8.99\times{10}^{3}$ |
| Initial value for extracellular HBcrAg for Mouse 604 | $R\left( 0 \right)$ | U/ml | $3.92\times{10}^{9}$ |
| **PEG IFN-α** |  |  |  |
| Initial value for extracellular HBV DNA for Mouse 501 | $V\left( 0 \right)$ | copies/ml | $9.26\times{10}^{9}$ |
| Initial value for extracellular HBsAg for Mouse 501 | $S\left( 0 \right)$ | IU/ml | $4.35\times{10}^{3}$ |
| Initial value for extracellular HBeAg for Mouse 501 | $E\left( 0 \right)$ | COI | $9.79\times{10}^{3}$ |
| Initial value for extracellular HBcrAg for Mouse 501 | $R\left( 0 \right)$ | U/ml | $4.49\times{10}^{9}$ |
| Initial value for extracellular HBV DNA for Mouse 502 | $V\left( 0 \right)$ | copies/ml | $2.29\times{10}^{9}$ |
| Initial value for extracellular HBsAg for Mouse 502 | $S\left( 0 \right)$ | IU/ml | $4.41\times{10}^{3}$ |
| Initial value for extracellular HBeAg for Mouse 502 | $E\left( 0 \right)$ | COI | $9.08\times{10}^{3}$ |
| Initial value for extracellular HBcrAg for Mouse 502 | $R\left( 0 \right)$ | U/ml | $3.81\times{10}^{9}$ |
| Initial value for extracellular HBV DNA for Mouse 503 | $V\left( 0 \right)$ | copies/ml | $3.66\times{10}^{9}$ |
| Initial value for extracellular HBsAg for Mouse 503 | $S\left( 0 \right)$ | IU/ml | $3.63\times{10}^{3}$ |
| Initial value for extracellular HBeAg for Mouse 503 | $E\left( 0 \right)$ | COI | $7.59\times{10}^{3}$ |
| Initial value for extracellular HBcrAg for Mouse 503 | $R\left( 0 \right)$ | U/ml | $3.69\times{10}^{9}$ |
| Initial value for extracellular HBV DNA for Mouse 504 | $V\left( 0 \right)$ | copies/ml | $5.03\times{10}^{9}$ |
| Initial value for extracellular HBsAg for Mouse 504 | $S\left( 0 \right)$ | IU/ml | $3.13\times{10}^{3}$ |
| Initial value for extracellular HBeAg for Mouse 504 | $E\left( 0 \right)$ | COI | $1.04\times{10}^{4}$ |
| Initial value for extracellular HBcrAg for Mouse 504 | $R\left( 0 \right)$ | U/ml | $3.22\times{10}^{9}$ |

**Table F. Quantified results for cccDNA in HBV infected mouse**

| **Experimental group A** | **cccDNA**^†^  **(band volume)** | **Average**  **(band volume)** | | **% of control** |
| --- | --- | --- | --- | --- |
| untreated control mouse A1 | $5.11\times{10}^{7}$ | $4.83\times{10}^{7}$ | $100$ | |
| untreated control mouse A2 | $4.55\times{10}^{7}$ | $-$ | $-$ | |
| PEG IFN-α treated mouse A1 | $1.74\times{10}^{7}$ | $1.60\times{10}^{7}$ | $33$ | |
| PEG IFN-α treated mouse A2 | $1.46\times{10}^{7}$ | $-$ | $-$ | |
| **Experimental group B** | **cccDNA**  **(band volume)** | **Average**  **(band volume)** | | **% of control** |
| untreated control mouse B1 | $1.31\times{10}^{7}$ | $1.13\times{10}^{7}$ | $100$ | |
| untreated control mouse B2 | $9.44\times{10}^{6}$ | $-$ | $-$ | |
| PEG IFN-α treated mouse B1 | $3.14\times{10}^{6}$ | $2.62\times{10}^{6}$ | $23$ | |
| PEG IFN-α treated mouse B2 | $2.10\times{10}^{6}$ | $-$ | $-$ | |

^†^cccDNA band volume was quantified from Southern blot data[^1^](#_ENREF_1). Briefly, mice infected with HBV at 12 weeks were treated with or without PEG IFN-α for 6 weeks, and then they were sacrificed. cccDNA levels were determined by Southern blot in Epicentre-based DNA extracts without proteinase K after PSD digestion. Experimental group A and B were performed as independent experiments.

**Note G: Transformation to a system of ODEs from a PDE multiscale model**

We here introduce a multiscale model using partial differential equations (PDEs) that couple intra-, inter- and extra-cellular virus dynamics for analyzing multiscale experimental data of HBV infection (c.f.[^2^](#_ENREF_2)) (**Fig. 2**):

$$\frac{dT\left( t \right)}{dt}=s-d_{T}T\left( t \right)-\beta T\left( t \right)V\left( t \right), \left( 5 \right)$$

$$\left( \frac{\partial}{\partial t}+\frac{\partial}{\partial a} \right)i\left( t,a \right)=-\delta i\left( t,a \right), \left( 6 \right)$$

$$\frac{dV(t)}{dt}=\left( 1-f \right)\rho\int_{0}^{\infty} D\left( t,a \right)i\left( t,a \right)da-\mu V(t), \left( 7 \right)$$

$$\frac{dS(t)}{dt}=\pi_{S}\int_{0}^{\infty} C\left( t,a \right)i\left( t,a \right)da+s_{i}\int_{0}^{\infty} i\left( t,a \right)da-\sigma S(t), \left( 8 \right)$$

$$\frac{dE\left( t \right)}{dt}=\pi_{E}\int_{0}^{\infty} C\left( t,a \right)i\left( t,a \right)da-\sigma E\left( t \right), \left( 9 \right)$$

$$\frac{dR\left( t \right)}{dt}=\pi_{R}\int_{0}^{\infty} C\left( t,a \right)i\left( t,a \right)da-\sigma R\left( t \right), \left( 10 \right)$$

$$\left( \frac{\partial}{\partial t}+\frac{\partial}{\partial a} \right)C\left( t,a \right)=f\rho D\left( t,a \right)-dC\left( t,a \right), \left( 11 \right)$$

$$\left( \frac{\partial}{\partial t}+\frac{\partial}{\partial a} \right)D\left( t,a \right)=\alpha C\left( t,a \right)-\rho D\left( t,a \right). \left( 12 \right)$$

As we recently reported,[^3^](#_ENREF_3)^,^[^4^](#_ENREF_4) the multiscale PDE model, Eqs.(5-12), can be transformed into a mathematically identical set of ordinary differential equations as follows. Using the method of characteristics with initial and boundary conditions of $i\left( t,a \right)$, we transform Eq. (6) into

$$i\left( t,a \right)=\left\{ \begin{aligned} e^{-\delta a}\beta T\left( t-a \right)V\left( t-a \right), t>a, \\ e^{-\delta t}i_{0}\left( a-t \right), t<a. \end{aligned} \right. \left( S1 \right)$$

Then, $I\left( t \right)$ is evaluated as follows:

$$I\left( t \right)=\int_{0}^{t} e^{-\delta a}\beta T\left( t-a \right)V\left( t-a \right)da+\int_{t}^{\infty} e^{-\delta t}i_{0}\left( a-t \right)da=\int_{0}^{t} e^{-\delta\left( t-a \right)}\beta T\left( a \right)V\left( a \right)da+\int_{0}^{\infty} e^{-\delta t}i_{0}\left( a \right)da.$$

Since $\frac{d}{dt}\int_{0}^{t} f\left( t,a \right)da=f\left( t,t \right)+\int_{0}^{t} \frac{\partial f\left( t,a \right)}{\partial t}da$, differentiating $I\left( t \right)$ with respect to time $t$, we obtain the following ODE:

$$\frac{dI(t)}{dt}=\beta T\left( t \right)V\left( t \right)-\delta I\left( t \right).$$

Also, we consider the total amount of cccDNA $CC(t)$ and the total amount of rcDNA $DD\left( t \right)$, defined by

$$CC\left( t \right)=\int_{0}^{\infty} C\left( t,a \right)i\left( t,a \right)da, DD\left( t \right)=\int_{0}^{\infty} D\left( t,a \right)i\left( t,a \right)da,$$

respectively. Here, under the biologically reasonable settings $C\left( t,0 \right)=0$, $D\left( t,0 \right)=1$ and $\lim_{a\to\infty} C\left( t,a \right)i\left( t,a \right)=\lim_{a\to\infty} D\left( t,a \right)i\left( t,a \right)=0$, we have

$$\frac{d}{dt}CC\left( t \right)=\int_{0}^{\infty} i\left( t,a \right)\frac{\partial}{\partial t}C\left( t,a \right)+C\left( t,a \right)\frac{\partial}{\partial t}i\left( t,a \right)da=f\rho DD\left( t \right)-(d+\delta)CC(t),$$

$$\frac{d}{dt}DD\left( t \right)=\int_{0}^{\infty} i\left( t,a \right)\frac{\partial}{\partial t}D\left( t,a \right)+D\left( t,a \right)\frac{\partial}{\partial t}i\left( t,a \right)da=\alpha CC\left( t \right)-\left( \rho+\delta\right)DD\left( t \right)+\beta T\left( t \right)V\left( t \right).$$

Therefore, the multiscale PDE model is described as the following equivalent system of ODEs:

$$\frac{dT\left( t \right)}{dt}=s-d_{T}T\left( t \right)-\beta T\left( t \right)V\left( t \right), \left( S2 \right)$$

$$\frac{dI\left( t \right)}{dt}=\beta T\left( t \right)V\left( t \right)-\delta I\left( t \right), \left( S3 \right)$$

$$\frac{dV\left( t \right)}{dt}=\left( 1-f \right)\rho DD\left( t \right)-\mu V\left( t \right), \left( S4 \right)$$

$$\frac{dS\left( t \right)}{dt}=\pi_{S}CC\left( t \right)+s_{i}I\left( t \right)-\sigma S\left( t \right), \left( S5 \right)$$

$$\frac{dE\left( t \right)}{dt}=\pi_{E}CC(t)-\sigma E\left( t \right), \left( S6 \right)$$

$$\frac{dR\left( t \right)}{dt}=\pi_{R}CC(t)-\sigma R\left( t \right), \left( S7 \right)$$

$$\frac{dCC\left( t \right)}{dt}=f\rho DD\left( t \right)-(d+\delta)CC(t), \left( S8 \right)$$

$$\frac{dDD(t)}{dt}=\alpha CC\left( t \right)-\left( \rho+\delta\right)DD\left( t \right)+\beta T\left( t \right)V\left( t \right). \left( S9 \right)$$

Note that Eqs. (*S*2-*S*9) will be further simplified for the purpose of data analysis depending on the antiviral treatment assumed (see later).

**Note H: Linearized equations under potent NAs treatment in humanized mouse**

We assumed that NAs treatment is potent enough that intracellular HBV replications and *de novo* infections are negligible after treatment initiation[^5-8^](#_ENREF_5) $\left( t=0 \right)$, i.e., the antiviral effectiveness of NAs on intracellular HBV replications is assumed to be $0<\varepsilon\leq1$and

$$i\left( t,a \right)=\left\{ \begin{matrix} 0, & t>a \\ e^{-\delta t}i_{0}\left( a-t \right), & t<a \end{matrix} \right..$$

Then Eqs. (*S*2-*S*9) can be simplified as follows:

$$\frac{dI\left( t \right)}{dt}=-\delta I\left( t \right), \left( S10 \right)$$

$$\frac{dV\left( t \right)}{dt}=\left( 1-f \right)\rho DD\left( t \right)-\mu V\left( t \right), \left( S11 \right)$$

$$\frac{dS\left( t \right)}{dt}=\pi_{S}CC\left( t \right)+s_{i}I\left( t \right)-\sigma S\left( t \right), \left( S12 \right)$$

$$\frac{dE\left( t \right)}{dt}=\pi_{E}CC(t)-\sigma E\left( t \right), \left( S13 \right)$$

$$\frac{dR\left( t \right)}{dt}=\pi_{R}CC(t)-\sigma R\left( t \right), \left( S14 \right)$$

$$\frac{dCC\left( t \right)}{dt}=f\rho DD\left( t \right)-(d+\delta)CC(t), \left( S15 \right)$$

$$\frac{dDD(t)}{dt}=(1-\varepsilon)\alpha CC\left( t \right)-\left( \rho+\delta\right)DD\left( t \right). \left( S16 \right)$$

Here we assume that all variables in Eqs. (*S*2-*S*9) are in steady state before treatment initiation[^9^](#_ENREF_9), and particularly that the infected cells obtain a stable age distribution, i.e., $i_{0}\left( a \right)=\beta T\left( 0 \right)V\left( 0 \right)e^{-\delta a}$.

Since Eqs. (*S*10-*S*16) are a set of linear ODEs, we directly solve them, and find the following analytical solutions:

$$V\left( t \right)=V\left( 0 \right)\left( Ae^{\left( \lambda_{1}-\delta\right)t}+Be^{\left( \lambda_{2}-\delta\right)t}+\left( 1-A-B \right)e^{-\mu t} \right), \left( S17 \right)$$

$$S\left( t \right)=S\left( 0 \right)\left( Ce^{\left( \lambda_{1}-\delta\right)t}+De^{\left( \lambda_{2}-\delta\right)t}+Ee^{-\delta t}+\left( 1-C-D-E \right)e^{-\sigma t} \right), \left( S18 \right)$$

$$E\left( t \right)=E\left( 0 \right)(C^{'}e^{\left( \lambda_{1}-\delta\right)t}+D^{'}e^{\left( \lambda_{2}-\delta\right)t}+\left( 1-C^{'}-D^{'} \right)e^{-\sigma t}), \left( S19 \right)$$

$$R\left( t \right)=R(0)(C^{'}e^{\left( \lambda_{1}-\delta\right)t}+D^{'}e^{\left( \lambda_{2}-\delta\right)t}+\left( 1-C^{'}-D^{'} \right)e^{-\sigma t}), \left( S20 \right)$$

moreover, the total amount of cccDNA $CC\left( t \right)$and the amount of cccDNA per infected cell $\tilde{C}\left( t \right)=CC(t)/I(t)$ are derived as follows:

$$CC\left( t \right)=CC\left( 0 \right)\left( Ze^{\left( \lambda_{1}-\delta\right)t}+\left( 1-Z \right)e^{\left( \lambda_{1}-\delta\right)t} \right), \left( S21 \right)$$

$$\tilde{C}\left( t \right)=\tilde{C}\left( 0 \right)\left( Ze^{\lambda_{1}t}+\left( 1-Z \right)e^{\lambda_{1}t} \right), \left( S22 \right)$$

where $A=\frac{-\left\{ {\left( \lambda_{1}+d+\delta\right)\lambda}_{2}+\delta\rho\right\}\mu}{\left( \lambda_{1}-\delta+\mu\right)(\lambda_{1}-\lambda_{2})\left( d+\delta\right)}, B=\frac{\left\{ {\left( \lambda_{2}+d+\delta\right)\lambda}_{1}+\delta\rho\right\}\mu}{\left( \lambda_{2}-\delta+\mu\right)(\lambda_{1}-\lambda_{2})\left( d+\delta\right)}$, $C=\frac{-\left( \lambda_{2}-\delta\right)\left( 1-x \right)\sigma}{\left( \lambda_{1}-\delta+\sigma\right)(\lambda_{1}-\lambda_{2})},$ $D=\frac{\left( \lambda_{1}-\delta\right)\left( 1-x \right)\sigma}{\left( \lambda_{2}-\delta+\sigma\right)(\lambda_{1}-\lambda_{2})}$, $E=\frac{\sigma x}{\sigma-\delta}$, $C^{'}=\frac{-(\lambda_{2}-\delta)\sigma}{\left( \lambda_{1}-\delta+\sigma\right)(\lambda_{1}-\lambda_{2})}, D^{'}=\frac{(\lambda_{1}-\delta)\sigma}{\left( \lambda_{2}-\delta+\sigma\right)(\lambda_{1}-\lambda_{2})}$, $Z=\frac{{-\lambda}_{2}+\delta}{\lambda_{1}-\lambda_{2}}$ and $\lambda_{1,2}=\frac{-\left( \rho+d \right)\pm\sqrt{\left( \rho-d \right)^{2}+4f\left( 1-\varepsilon\right)\alpha\rho}}{2}$. Note that $x$ is the proportion of HBsAg produced from integrated DNA: $x=\frac{s_{i}I\left( 0 \right)}{\pi CC\left( 0 \right)+s_{i}I\left( 0 \right)}.$

**Note I: Linearized equations under potent PEG IFN-α treatment in humanized mouse**

We also assumed that PEG IFN-α treatment is potent enough that intracellular HBV replication and *de novo* infections are negligible after treatment initiation[^6^](#_ENREF_6)^,^[^7^](#_ENREF_7)^,^[^10-12^](#_ENREF_10) $\left( t=0 \right)$, i.e., the antiviral effect of PEG IFN-α on intracellular HBV replications is assumed to be $0<\varepsilon\leq1$and

$$i\left( t,a \right)=\left\{ \begin{matrix} 0, & t>a \\ e^{-\delta_{INF}t}i_{0}\left( a-t \right), & t<a \end{matrix} \right..$$

Then Eqs. (*S*2-*S*9) can be simplified to

$$\frac{dI\left( t \right)}{dt}=-\delta_{IFN}I\left( t \right), \left( S23 \right)$$

$$\frac{dV\left( t \right)}{dt}=\left( 1-f \right)\rho DD\left( t \right)-\mu V\left( t \right), \left( S24 \right)$$

$$\frac{dS\left( t \right)}{dt}=\pi_{S}CC\left( t \right)+s_{i}I\left( t \right)-\sigma S\left( t \right), \left( S25 \right)$$

$$\frac{dE\left( t \right)}{dt}=\pi_{E}CC(t)-\sigma E\left( t \right), \left( S26 \right)$$

$$\frac{dR\left( t \right)}{dt}=\pi_{R}CC(t)-\sigma R\left( t \right), \left( S27 \right)$$

$$\frac{dCC\left( t \right)}{dt}=f\rho DD\left( t \right)-(d_{IFN}+\delta_{IFN})CC(t), \left( S28 \right)$$

$$\frac{dDD(t)}{dt}=(1-\varepsilon)\alpha CC\left( t \right)-\left( \rho+\delta_{IFN} \right)DD\left( t \right). \left( S29 \right)$$

In addition, it has been reported that PEG IFN-α induces interferon-stimulated genes (ISGs) and ISGs potentially degrade intracellular cccDNA. Therefore, we assumed PEG IFN-α increases the cccDNA degradation rate[^13^](#_ENREF_13), i.e., $d_{IFN} (>d)$. Similarly, we assume that all variables in Eqs. (*S*2-*S*9) are in steady state before treatment initiation, and that the infected cells have obtained a stable age distribution, i.e., $i_{0}\left( a \right)=\beta T\left( 0 \right)V\left( 0 \right)e^{-\delta a}$. Because PEG IFN-α may enhance the decay rate of infected cells in HBV infection due to cytotoxic effects (but relatively mild), we assumed $\delta_{IFN} (\geq\delta)$ in the data fitting (**Fig 3AB** and **Fig C in S1 Text**). Solving Eqs. (*S*21-*S*27) we find

$$V\left( t \right)=V\left( 0 \right)\left( A_{IFN}e^{\left( \eta_{1}-\delta_{IFN} \right)t}+B_{IFN}e^{\left( \eta_{2}-\delta_{IFN} \right)t}+\left( 1-A_{IFN}-B_{IFN} \right)e^{-\mu t} \right), \left( S30 \right)$$

$$S\left( t \right)=S\left( 0 \right)\left( C_{IFN}e^{\left( \eta_{1}-\delta_{IFN} \right)t}+D_{IFN}e^{\left( \eta_{2}-\delta_{IFN} \right)t}+E_{IFN}e^{-\delta_{IFN}t}+\left( 1-C_{IFN}-D_{IFN}-E_{IFN} \right)e^{-\sigma t} \right), \left( S31 \right)$$

$$E\left( t \right)=E\left( 0 \right)\left( C_{IFN}^{'}e^{\left( \eta_{1}-\delta_{IFN} \right)t}+D_{IFN}^{'}e^{\left( \eta_{2}-\delta_{IFN} \right)t}+\left( 1-C_{IFN}^{'}-D_{IFN}^{'} \right)e^{-\sigma t} \right), \left( S32 \right)$$

$$R\left( t \right)=R\left( 0 \right)\left( C_{IFN}^{'}e^{\left( \eta_{1}-\delta_{IFN} \right)t}+D_{IFN}^{'}e^{\left( \eta_{2}-\delta_{IFN} \right)t}+\left( 1-C_{IFN}^{'}-D_{IFN}^{'} \right)e^{-\sigma t} \right), \left( S33 \right)$$

moreover, the total amount of cccDNA $CC\left( t \right)$and the amount of cccDNA per infected cell $\tilde{C}\left( t \right)=CC(t)/I(t)$ are derived as follows

$$CC\left( t \right)=CC\left( 0 \right)\left( Z_{IFN}e^{\left( \eta_{1}-\delta_{IFN} \right)t}+\left( 1-Z_{IFN} \right)e^{\left( \eta_{2}-\delta_{IFN} \right)t} \right), \left( S34 \right)$$

$$\tilde{C}\left( t \right)=\tilde{C}\left( 0 \right)\left( Z_{IFN}e^{\eta_{1}t}+\left( 1-Z_{IFN} \right)e^{\eta_{2}t} \right), \left( S35 \right)$$

where $A_{IFN}=\frac{-\left\{ {\left( \eta_{1}+d+\delta\right)\eta}_{2}+\left( d-d_{IFN}+\delta\right)\rho\right\}\mu}{\left( \eta_{1}-\delta_{IFN}+\mu\right)(\eta_{1}-\eta_{2})\left( d+\delta\right)}, B_{IFN}=\frac{\left\{ {\left( \eta_{2}+d+\delta\right)\eta}_{1}+\left( d-d_{IFN}+\delta\right)\rho\right\}\mu}{\left( \eta_{2}-\delta_{IFN}+\mu\right)(\eta_{1}-\eta_{2})\left( d+\delta\right)}, C_{IFN}=\frac{-\left( \eta_{2}-d+d_{IFN}-\delta\right)\left( 1-x \right)\sigma}{\left( \eta_{1}-\delta_{IFN}+\sigma\right)(\eta_{1}-\eta_{2})}, D_{IFN}=\frac{\left( \eta_{1}-d+d_{IFN}-\delta\right)\left( 1-x \right)\sigma}{\left( \eta_{2}-\delta_{IFN}+\sigma\right)(\eta_{1}-\eta_{2})}, E_{IFN}=\frac{\sigma x}{\sigma-\delta_{IFN}}, C_{IFN}^{'}=\frac{-\left( \eta_{2}-d+d_{IFN}-\delta\right)\sigma}{\left( \eta_{1}-\delta_{IFN}+\sigma\right)(\eta_{1}-\eta_{2})}, D_{IFN}^{'}=\frac{\left( \eta_{1}-d+d_{IFN}-\delta\right)\sigma}{\left( \eta_{2}-\delta_{IFN}+\sigma\right)(\eta_{1}-\eta_{2})} ,Z_{IFN}=\frac{{-\eta}_{2}+d-d_{IFN}+\delta}{\eta_{1}-\eta_{2}}$and $\eta_{1,2}=\frac{-\left( d_{IFN}+\rho\right)\pm\sqrt{\left( d_{IFN}-\rho\right)^{2}+4f\left( 1-\varepsilon\right)\alpha\rho}}{2}$. Note that $x$ is the proportion of HBsAg produced from integrated DNA: $x=\frac{s_{i}I\left( 0 \right)}{\pi CC\left( 0 \right)+s_{i}I\left( 0 \right)}.$

**References**

1. Allweiss, L.*, et al.* Therapeutic shutdown of HBV transcripts promotes reappearance of the SMC5/6 complex and silencing of the viral genome in vivo. *Gut* (2021).

2. Iwanami, S.*, et al.* Should a viral genome stay in the host cell or leave? A quantitative dynamics study of how hepatitis C virus deals with this dilemma. *PLoS Biol* **18**, e3000562 (2020).

3. Kitagawa, K.*, et al.* Mathematical Analysis of a Transformed ODE from a PDE Multiscale Model of Hepatitis C Virus Infection. *Bull Math Biol* **81**, 1427-1441 (2019).

4. Kitagawa, K., Nakaoka, S., Asai, Y., Watashi, K. & Iwami, S. A PDE multiscale model of hepatitis C virus infection can be transformed to a system of ODEs. *J Theor Biol* **448**, 80-85 (2018).

5. Alonso, S.*, et al.* Upcoming pharmacological developments in chronic hepatitis B: can we glimpse a cure on the horizon? *BMC Gastroenterol* **17**, 168 (2017).

6. Fatehi, F., Bingham, R.J., Stockley, P.G. & Twarock, R. An age-structured model of hepatitis B viral infection highlights the potential of different therapeutic strategies. *Sci Rep* **12**, 1252 (2022).

7. Goyal, A., Liao, L.E. & Perelson, A.S. Within-host mathematical models of hepatitis B virus infection: Past, present, and future. *Curr Opin Syst Biol* **18**, 27-35 (2019).

8. Wolters, L.M., Hansen, B.E., Niesters, H.G., DeHertogh, D. & de Man, R.A. Viral dynamics during and after entecavir therapy in patients with chronic hepatitis B. *J Hepatol* **37**, 137-144 (2002).

9. Neumann, A.U.*, et al.* Hepatitis C viral dynamics in vivo and the antiviral efficacy of interferon-alpha therapy. *Science* **282**, 103-107 (1998).

10. Colombatto, P.*, et al.* A multiphase model of the dynamics of HBV infection in HBeAg-negative patients during pegylated interferon-alpha2a, lamivudine and combination therapy. *Antivir Ther* **11**, 197-212 (2006).

11. Ribeiro, R.M.*, et al.* Hepatitis B virus kinetics under antiviral therapy sheds light on differences in hepatitis B e antigen positive and negative infections. *J Infect Dis* **202**, 1309-1318 (2010).

12. Reinharz, V.*, et al.* Understanding Hepatitis B Virus Dynamics and the Antiviral Effect of Interferon Alpha Treatment in Humanized Chimeric Mice. *J Virol* **95**, e0049220 (2021).

13. Lucifora, J.*, et al.* Specific and nonhepatotoxic degradation of nuclear hepatitis B virus cccDNA. *Science* **343**, 1221-1228 (2014).
